# Eye Infections Caused by Filamentous Fungi: Spectrum and Antifungal Susceptibility of the Prevailing Agents in Germany

**DOI:** 10.3390/jof7070511

**Published:** 2021-06-26

**Authors:** Grit Walther, Anna Zimmermann, Johanna Theuersbacher, Kerstin Kaerger, Marie von Lilienfeld-Toal, Mathias Roth, Daniel Kampik, Gerd Geerling, Oliver Kurzai

**Affiliations:** 1 National Reference Center for Invasive Fungal Infections (NRZMyk), Leibniz Institute for Natural Product Research and Infection Biology—Hans Knöll Institute, Adolf-Reichwein-Str. 23, 07745 Jena, Germany; kkaerger@web.de (K.K.); Marie.von_Lilienfeld-Toal@med.uni-jena.de (M.v.L.-T.); okurzai@hygiene.uni-wuerzburg.de (O.K.); 2 Institute for Hygiene and Microbiology, University of Würzburg, 97080 Würzburg, Germany; anna.zimmermann@uni-wuerzburg.de; 3 Department of Ophthalmology, University Hospital Würzburg, 97080 Würzburg, Germany; j.theuersbacher@icloud.com (J.T.); Kampik_D@ukw.de (D.K.); 4 Department of Haematology and Medical Oncology, University Hospital Jena, 07747 Jena, Germany; 5 Department of Ophthalmology, Heinrich-Heine University Düsseldorf, 40225 Düsseldorf, Germany; mathiasroth@gmx.net (M.R.); geerling@med.uni-duesseldorf.de (G.G.)

**Keywords:** eye infection, fungal infection, keratitis, antifungal susceptibility, natamycin, *Fusarium*, *Purpureocillium*, *Aspergillus*, *Alternaria*, *Scedosporium*

## Abstract

Fungal eye infections can lead to loss of vision and blindness. The disease is most prevalent in the tropics, although case numbers in moderate climates are increasing as well. This study aimed to determine the dominating filamentous fungi causing eye infections in Germany and their antifungal susceptibility profiles in order to improve treatment, including cases with unidentified pathogenic fungi. As such, we studied all filamentous fungi isolated from the eye or associated materials that were sent to the NRZMyk between 2014 and 2020. All strains were molecularly identified and antifungal susceptibility testing according to the EUCAST protocol was performed for common species. In total, 242 strains of 66 species were received. *Fusarium* was the dominating genus, followed by *Aspergillus*, *Purpureocillium*, *Alternaria*, and *Scedosporium*. The most prevalent species in eye samples were *Fusarium petroliphilum*, *F. keratoplasticum*, and *F. solani* of the *Fusarium solani* species complex. The spectrum of species comprises less susceptible taxa for amphotericin B, natamycin, and azoles, including voriconazole. Natamycin is effective for most species but not for *Aspergillus flavus* or *Purpureocillium* spp. Some strains of *F. solani* show MICs higher than 16 mg/L. Our data underline the importance of species identification for correct treatment.

## 1. Introduction

Eye infections caused by fungi are serious diseases that can lead to loss of vision and blindness. Sites of exogenous infections mainly include the cornea (keratitis), but also the vitreous body (endophthalmitis), often as consequence of progressing keratitis. According to estimates, more than one million people are affected by fungal keratitis annually and 8–11% of the patients lose the eye [1]. The disease is most prevalent in tropical and subtropical countries [2,3], but an increasing number of cases has also been reported in countries with moderate climates [4,5,6,7,8,9,10]. Numerous fungal species are known to cause keratitis. Clinically, wearing of soft contact lenses and ocular trauma is mostly associated with keratitis caused by filamentous fungi, whereas keratitis caused by *Candida* spp. most frequently occurs in patients with chronic eye disease, topical steroid use, or surgical intervention. The spectrum of filamentous fungi causing keratitis differs depending on the climate and geographic region, but *Aspergillus* and *Fusarium* species are the predominant causative agents worldwide [11,12,13]. In several countries, including India, Nepal, and the USA (Florida), *Curvularia* species play an important role as the cause of keratitis [12]. Data from the German Fungal Keratitis Registry have shown that roughly one-third of cases in Germany are caused by *Candida* species, one-third is *Fusarium* keratitis, and one-third is caused by other filamentous fungi, including *Aspergillus* spp. [14].

In the two most frequently isolated genera of filamentous fungi associated with eye infections, *Fusarium* and *Aspergillus*, numerous new species have been described in the last years based on DNA sequence data [15,16,17,18,19]. As a consequence, morphology based approaches often only allow the identification of species complexes or sections, respectively. Even molecular species identification by sequencing of the nuclear ribosomal internal transcribed spacer (ITS) region that is used as a universal marker in fungi [20] cannot discriminate all species in these genera [21,22]. Consequently, exact species identification is often lacking in published case series of keratitis caused by filamentous fungi. 

The taxonomy and nomenclature of the genus *Fusarium* are currently controversial. Some authors favor the use of the name *Fusarium* and a wide generic concept including the FSSC [23], while other authors support a smaller generic concept that splits the genus *Fusarium* into several genera (e.g., *Neocosmospora*), primarily based on characteristics of their fruiting bodies (perithecia), but also on the asexual morphs [24]. Due to the fact that *Fusarium* is of great importance in medical mycology because it is one of the opportunistic fungal genera that is recognized morphologically, we use the first concept and the nomenclature suggested by Geiser et al. [23].

Currently, the treatment of fungal keratitis is mainly based on the polyene drugs natamycin (also known as pimaricin) or amphotericin B and the azole antifungal voriconazole. All three drugs can be applied topically, whereas evidence suggests that only voriconazole should be considered for systemic treatment [25]. Natamycin and amphotericin B largely have good in vitro activity against *Fusarium* spp. [26]. Voriconazole is clinically effective against *Fusarium* spp., despite variable in vitro activity. It is effective against some species of *Aspergillus* [27] and *Scedosporium* [28]. Local natamycin was associated with better visual acuity after infection and a reduced number of corneal perforations or the need to perform therapeutic keratoplasty compared to monotherapy by voriconazole [25].

To assess the epidemiological situation of keratitis caused by filamentous fungi in Germany and provide representative data on the in vitro antifungal susceptibility of the causative agents, this study aims to answer the following questions: (1) Which filamentous fungal species are causing keratitis in Germany? (2) What are the antifungal susceptibility profiles of these species? (3) Is natamycin effective against all fungal taxa causing eye infections? (4) Are there differences in the minimum inhibitory concentrations (MICs) of natamycin among *Fusarium* species?

To answer these questions, we studied all filamentous fungi from eye infections that were sent to the National Reference Center for Invasive Fungal Infections (NRZMyk) between January 2014 and December 2020. Molecular species identification was performed for all isolates using reliable markers established for the respective genera. Antifungal susceptibility was tested in vitro according to the EUCAST protocol for all *Fusarium* strains, as well as for other more frequent species.

On the basis of molecular species identifications, we showed that the prevalent agents of eye infections in Germany belong to the genera *Fusarium*, *Aspergillus*, *Purpureocillium*, *Alternaria*, and *Scedosporium*. The antifungal susceptibility profiles revealed that natamycin is effective for most species but not for *Aspergillus flavus* or the genus *Purpureocillium*, representing common agents of eye infections in Germany. Within the genus *Fusarium*, only some *F. solani* isolates showed MICs > 16 mg/L. 

## 2. Material and Methods

### 2.1. Isolates

Filamentous fungi isolated from the eye (corneal and conjunctival swabs and scrapings, aspirates of the anterior chamber or the vitreous body) or from eye-associated materials such as contact lenses or cleaning solutions that were sent to the NRZMyk between January 2014 and December 2020 were included in the study of the spectrum of species involved in eye infections. Due to research activities in this area, the NRZMyk receives a high number of samples from cases of filamentous keratitis. In case several isolates of the same species from the same patient were received, only the initial isolate was included. For antifungal susceptibility testing (AFST), additional strains from other sources were included for species represented by a small number of eye-related isolates in order to cover the variability of the respective species. Isolates related to ocular infections, as well as isolates from other sources that were used for AFST, were deposited in the Jena Microbial Resource Collection (JMRC). Strain numbers, GenBank accession numbers of the identifying sequences, sources, and the minimum inhibitory concentrations are given in Table S1.

### 2.2. Molecular Species Identification

Genomic DNA was extracted from 2- to 7-day-old cultures grown on 4% malt extract agar (Difco), as described before [9]. Depending on the genus, different markers were amplified by PCR for species identification. Table S2 provides the primers used for PCR and sequencing of each genus, as well as their annealing temperatures. The SeqMan program v. 11.0.0. (DNASTAR, Lasergene, Madison, WI, USA) was used to construct consensus sequences. Species were identified by using the BLAST tool in GenBank (Available online: blast.ncbi.nlm.nih.gov/Blast.cgi?PAGE_TYPE=BlastSearch, accessed on 31 March 2021). Sequences were deposited at GenBank (Available online: www.ncbi.nlm.nih.gov/genbank/, accessed on 31 March 2021) and their accession numbers are listed in Table S1. 

### 2.3. Antifungal Susceptibility Testing

In vitro antifungal susceptibilities of all *Fusarium* species and all other species that were isolated at least three times from infected eyes or eye-associated material were performed by broth microdilution technique following the European Committee on Antimicrobial Susceptibility Testing (EUCAST) standard methodology [29]. If the number of isolates from eyes or eye-associated material was below 10 for a species, additional antifungal susceptibility tests were performed with isolates of this species from other sources for a better coverage of the variability of the species (Table S1). The following antifungals were tested: amphotericin B (AMB; European Pharmacopoeia, Strasbourg, France); caspofungin (CAS; MSD, Rahway, NJ, USA), isavuconazol (ISA; Basilea Pharmaceutica International Ltd., Basel, Switzerland); itraconazole (ITZ), natamycin (NAT) (Chemicalpoint, Deisenhofen, Germany); posaconazole (PCZ; MSD, Rahway, NJ, USA); and voriconazole (VCZ; Pfizer Inc., Peapack, NJ, USA). 

Fungi were grown on MEA for 2 to 7 days at 35 °C (*Aspergillus*), 30 °C (*Alternaria*, *Fusarium*, *Scedosporium*), or room temperature (*Cladosporium*). Spore suspensions were counted with a hemocytometer. MIC endpoints were defined as 100% reduction in growth and were determined visually using a mirror after 48 h of incubation at 35 °C, except for *Cladosporium* strains, for which the endpoints were determined after 72 h at 30 °C due to their lower maximum temperature of growth. For caspofungin, minimum effective concentrations (MECs) were determined by reading the microplates with the aid of an inverted microscope. *Aspergillus fumigatus* ATCC 204305 and *Candida parapsilosis* ATCC 22019 served as reference strains. For the calculation of geometric means, high off-scale MICs/MECs were raised to the next higher concentration.

## 3. Results

### 3.1. Spectrum of Filamentous Fungi Causing Eye Infections in Germany

In the 7-year period from 2014 to 2020, the NRZMyk received 242 strains of 66 species related to eye infections of 234 patients. In six cases, we received two different species from the same patient, while in a single case we received three different species isolated from the same patient. Of the 66 species received in total, 35 species were exclusively isolated from the eye, 17 species were isolated from the eye and eye-associated material, and 14 species were isolated from eye-associated material only (Table 1 and Table S1).

With few exceptions we were able to identify the strains molecularly by the markers listed in Table S2. One *Fusarium* strain (FDSC) could not be identified at the species level due to a lack of reference sequences in GenBank. For one strain of *Plectosphaerella* sp. and one strain of *Pseudopithomyces* sp., the ITS region was not discriminative enough to identify the species. 

**Figure 1:**
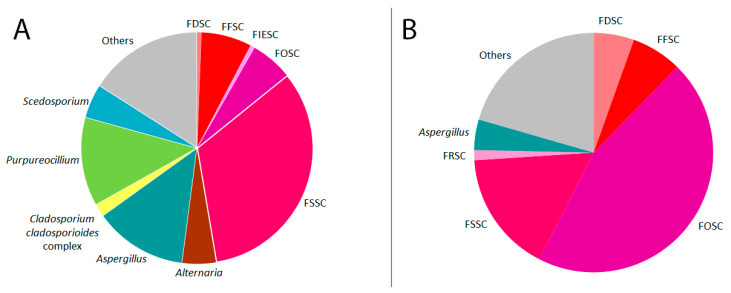
Taxa of filamentous fungi isolate from infected eyes (**A**) or eye-associated materials (**B**). Species complexes (sc) of *Fusarium* are highlighted in shades of red: *Fusarium dimerum* sc (FDSC), *F. fujikuroi* sc (FFSC), *F. incarnatum-equiseti* sc (FIESC), *F. oxysporum* sc (FOSC), *F. redolens* sc (FRSC), *F. solani* sc (FSSC). Genera that are presented by only one or two isolates are shown in the “Others” section of the pie chart.

Among the isolates from the eye, *Fusarium* is the dominant genus (80 strains, 47.3%), followed by *Aspergillus* (22 strains, 13.0%), *Purpureocillium* (21 strains, 12.4%), *Alternaria* (8 strains, 4.7%), and *Scedosporium* (8 strains, 4.7%) (Figure 1). Within the genus *Fusarium*, the *Fusarium solani* species complex (FSSC) is predominant, while the *Fusarium fujikuroi* species complex (FFSC) is the second most common and the *Fusarium oxysporum* species complex (FOSC) is the third most common (Table 1). With 8 species isolated from the eye, the FSSC is represented by the highest number of species. The most prevalent FSSC species are *F. petroliphilum*, *F. keratoplasticum*, and *F. solani*. Predominant species in the remaining genera are *Purpureocillium lilacinum*, *Aspergillus fumigatus*, *A. flavus*, *Alternaria alternata*, and *Scedosporium apiospermum*. Beside *Purpureocillium lilacinum*, the recently described sibling species *Purpureocillium sodanum* was identified in 6 cases.

*Fusarium* is also the dominant genus among isolates from eye-associated materials (75.3%). Isolates of *Aspergillus* and *Purpureocillum* that make up high proportions of isolates from the eye are only rarely or not isolated from eye-associated materials (Table 1, Figure 1). In *Fusarium*, most of the species that are commonly isolated from the eye, such as *F. petroliphilum* or *F. solani*, were also isolated from eye-associated materials in higher proportions than other species, but in numbers that were distinctly lower than those from the eye. An exception was the FOSC. In this complex, the number of isolates from eye-associated materials was more than three times higher than the number of isolates from the eye (Table 1, Figure 1).

### 3.2. Antifungal Susceptibility Testing Profiles of Eye-Infecting Fungal Species

In total, AFST was performed for 257 strains of the 33 species that are considered to be eye pathogens, because they were repeatedly isolated from the infected eye. Strains isolated from eye samples and related materials exhibit similar profiles to strains from other sources (Table S1). The AFST profiles of the species causing eye infections differ strongly. None of the tested antifungals is effective against all species studied: species of the genera *Purpureocillium*, *Lomentospora*, *Scedosporium*, and *Scopulariopsis* show high MICs for amphotericin B; *Aspergillus flavus* and *Purpureocillium* spp. show high MICs for natamycin; while *Fusarium* species, especially of the FSSC, exhibit high MICs for isavuconazole, itraconazole, posaconazole, voriconazole, and caspofungin (Table 2 and Table 3). 

Amphotericin B and natamycin are both polyene antifungals. However, high MICs for amphotericin B did not necessarily correspond to high natamycin MICs in all taxa. Species of *Scedosporium*, *Lomentospora prolificans*, and *Scopulariopsis brevicaulis* have high MICs for amphotericin B but low MICs for natamycin (Table 2). In contrast, *Purpureocillium* spp. show high MICs for amphotericin B and for natamycin (Table 2). While this may suggest differential activity of the two agents, it remains unclear how this translates into clinical treatment response.

Within the genus *Fusarium*, the studied species complexes did not differ regarding their susceptibility to amphotericin B, natamycin, itraconazole, and caspofungin (Table 2 and Table 3). In vitro, amphotericin B was the most effective drug. The MICs for natamycin ranged between 2 and 8 mg/L, except for *F. solani*, with natamycin MICs of up to 32 mg/L. Species of the FSSC had high MICs for the azoles tested (ISA, ITZ, PCZ, VCZ), with the lowest MICs found for voriconazole. Slightly lower MICs for voriconazole were found in the FOSC. The species of FFSC possessed specific profiles—*F. musae*, *F. sacchari*, and *F. verticillioides* were more susceptible to azoles, especially posaconazole and voriconazole, while *F. proliferatum* and *F. lactis* had MICs similar to the FSSC (Table 2 and Table 3).

*Purpureocillium lilacinum* and *P. sodanum*, as well as *Tintelnotia destructans*, had characteristic AFS profiles for azoles. The two *Purpureocillium* species showed high MICs for itraconazole but low MICs for isavuconazole, posaconazole, and voriconazole, while *Tintelnotia destructans* exhibited high MICs for isavuconazole but low MICs for itraconazole, posaconazole, and voriconazole.

All three studied species of *Scedosporium* showed low MICs for voriconazole, in contrast to the closely related *Lomentospora prolificans*, which showed high MICs for voriconazole as well (Table 2 and Table 3).

## 4. Discussion

This study presents the first spectrum of filamentous fungi isolated from infected eyes that are molecularly identified by the use of the ID markers established in the respective genera. Sample submission to the NRZMyk is non-systematic and likely biased towards clinically relevant isolates and isolates that are not easily identified.

Fifty-two species were isolated from the eye and eye-associated materials, while 14 species were restricted to eye-associated materials only. *Chaetomium anastomosans*, *Coprinellus domesticus* (syn. *Hormographiella verticillata*), *Eppicoccum mezzettii*, *Fusarium stercicola*, *Lecanicillium coprophilum*, *Penicillium crustosum*, *Petriella setifera*, *Purpureocillium sodanum*, *Rhinocladiella similis*, and *Sarocladium spinificis* have not been reported to cause eye infections. *Chaetomium anastomosans* (sibling species of *C. globosum)* [30] and *Purpureocillium sodanum* (sibling species of *P. lilacinum)* [31] are recently described species, cases of which might have previously been assigned to their sibling species. Other species such as *Aspergillus cibarius* [32], *A. udagawae* [33], *Fusarium bostrycoides* (as FSSC 25), *F. tonkinense* (as FSSC 9) [9], *Penicillium rubens* [34], and *Scedosporium dehoogii* [35] have been reported only once before in connection with eye infections.

Among all filamentous fungi associated with eye infections, *Fusarium* is the dominating genus, at nearly 47.3%. In the genus *Fusarium*, species of the FSSC (*F. petroliphilum*, *F. keratoplasticum*, and *F. solani*) are most frequently isolated from the infected eye, in agreement with former studies [9,36,37,38,39]. Studies in France and the Netherlands found higher proportions of the FFSC (47%) and the FOSC (41%) [40] or the FOSC (24.7%) [10], respectively. FOSC from contact lenses and their cleaning solution suggests that this species is a frequent contaminant of eye-associated materials, which is supported by a previous case study [9]. Some studies have shown a lower pathogenic potential of the FOSC compared to the FSSC [41,42,43]. On the other hand, this observation could mean that the cultivation of the FOSC from infected tissue fails more often, e.g., because its viability is more affected by antifungal treatment.

Compared to species of *Aspergillus* (5.5%) and *Purpureocillium* (0%), *Fusarium* spp. were more frequently isolated from contact lenses or their cleaning solutions (75.3% in total: FOSC 45.2%, remaining *Fusarium* species 30.1%; Table 1, Figure 1). One reason could be that *Aspergillus* spp. and *Purpureocillium* spp. grow better from eye samples, making isolations from eye-associated materials unnecessary. Another reason could be that the antimicrobial agents of the cleaning solutions are less effective against *Fusarium* species; thus, the reduced activity of alexidine in contact lens cleaning solutions after heating caused a worldwide outbreak of Fusarium keratitis, while cases of keratitis caused by other fungal genera did not increase markedly [44,45].

Other important eye-infecting genera are *Aspergillus* (13.0%), *Purpureocillium* (12.4%), *Alternaria* (4.7%), and *Scedosporium* (4.7%). Strikingly, dematiaceous genera such as *Alternaria* and *Cladosporium* contribute to a smaller extent compared to studies of tropical and subtropical regions with more intensive UV radiation [2,12,36,46,47,48,49,50]. Dematiaceous genera such as *Bipolaris* and *Curvularia*, which are important eye pathogens in the tropics and subtropics, were not sent to the NRZMyk.

The high proportion of *Purpureocillium* species in eye infections in Germany is remarkable. *Purpureocillium lilacinum* used to be named *Paecilomyces lilacinus*. *Paecilomyces* spp. are mentioned as causative agents in several studies of fungal keratitis [46,48,49], but the percentage of the *Purpureocillium* species included is unknown. In the Assam region of North India, *Purpureocillium lilacinum* is causative in 1.6% of fungal keratitis cases [51], while in the USA it accounts for 4.4% [52]. One of the major risk factors for infections by *P. lilacinum* is the use of contact lenses [52,53], which might explain its high proportion in Germany, where wearing contact lenses is popular [54]. *Purpureocillium lilacinum* and its sibling species *P. sodanum* [31] are morphologically similar but their ITS sequences differ only by a single base pair. As a consequence, it is more likely that *P. sodanum* has been overlooked than that it is emerging.

The important keratitis-causing fungi in Germany show diverse antifungal susceptibility profiles and include taxa with high MICs for all antifungals tested. Amphotericin B was the antifungal agent with the lowest MICs for *Fusarium* spp. and with low MICs for several other species (Table 2 and Table 3); however, eye pathogens such as *Purpureocillium* spp., *Lomentospora prolificans*, *Scedosporium* spp., and *Scopulariopsis brevicaulis* exhibit high amphotericin B MICs, which is in agreement with previous studies [28,55,56,57,58,59]. Natamycin MICs are usually higher compared to amphotericin B MICs, but isolates with natamycin MICs of ≤16 mg/L are considered susceptible because this concentration is reached in the eye during therapy [60,61]. The ineffectiveness of natamycin for *Aspergillus flavus* and *Purpureocillium* spp. has been reported previously [52,55].

Some of the *Fusarium solani* strains tested in our study had reduced susceptibility for natamycin (MIC > 16 mg/L). Although members of the FSSC exhibit similar AFS profiles, we found MIC > 16 mg/L only for this species of the FSSC. In a Dutch study, MICs of 16 mg/L for some strains of *F. solani* and *F. falciforme* were observed [62]. An Indian study found natamycin MICs for the FSSC ranging between 8–128 mg/L [55]. To date, we do not know if these results of in vitro tests have an impact on the outcome of *F. solani* infections, but in keratitis cases caused by this species that do not respond to natamycin, a switch of the therapeutic agent should be considered.

The genera included in our study showed different susceptibilities concerning the polyenes—*Purpureocillium* spp. were not susceptible to amphotericin B or natamycin, *Aspergillus flavus* showed reduced MICs for amphotericin B but high MICs for natamycin and *Scedosporium* spp., *Lomentospora prolificans* and *Scopulariopsis brevicaulis* exhibited normal MICs for natamycin but high MICs for amphotericin B. These results are in concordance with the finding that natamycin has a different mode of action. As all polyene drugs, natamycin binds to ergosterol; however, in contrast to amphotericin B, it does not change the permeability of the plasma membrane, resulting in the leakage of essential components; rather, it impairs the membrane fusion [63,64].

The susceptibility levels for azoles determined in this study show differences among the species complexes that are in agreement with former studies [65,66,67], with high MICs for all azoles, including voriconazole in the FSSC; slightly lower MICs for voriconazole in the FOSC; and lower MICs for isavuconazole, posaconazole and voriconazole for most of the FFSC species. One exception to this is *Fusarium proliferatum*, which also had high MICs for isavuconazole, posaconazole, and voriconazole, although it belongs to the FFSC. By using the CLSI protocol, lower posaconazole MICs were found for this species [26].

In conclusion, in Germany the predominant filamentous fungi infecting eyes belong to the genera *Fusarium*, *Aspergillus*, *Purpureocillium*, *Alternaria*, and *Scedosporium*. Differences in their AFS profiles, which include high MICs for all important antifungals in keratitis treatment (natamycin, amphotericin B, voriconazole), favor combined therapy and underline the importance of the identification of the aetiological agent. Some strains of *Fusarium solani* exhibited natamycin MICs > 16 mg/L. Although amphotericin B and natamycin are both polyenes, the levels of MIC values of amphotericin B of a certain species are not predictive of MIC values of natamycin, and vice versa.

## Figures and Tables

**Table 1:** Spectrum of fungi isolated from infected eyes and associated materials (corneal and conjunctival swabs, aspirates of the anterior chamber and the vitreous body). For the FSSC, the numbering of the phylogenetic species is given in brackets.

Species	No. of Isolates from Eye	No. of Isolates from Eye-Associated Materials
**Total strain number**	**169 (100%)**	**73 (100%)**
***Alternaria***	**8 (4.7%)**	**2 (2.7%)**
*Alternaria alternata*	8 (4.7%)	0
*Alternaria hordeicola*	0	1 (1.4%)
*Alternaria rosae*	0	1 (1.4%)
*Arthrographis kalrae*	2 (1.2%)	0
***Aspergillus***	**22 (13.0%)**	**4 (5.5%)**
*Aspergillus cibarius*	1 (0.6%)	0
*Aspergillus flavus*	6 (3.6%)	1 (1.4%)
*Aspergillus fumigatus*	10 (5.9%)	1 (1.4%)
*Aspergillus hiratsukae*	1 (0.6%)	0
*Aspergillus sydowii*	0	1 (1.4%)
*Aspergillus terreus*	1 (0.6%)	0
*Aspergillus tubingensis*	2 (1.2%)	1 (1.4%)
*Aspergillus udagawae*	1 (0.6%)	0
*Aureobasidium pullulans*	1 (0.6%)	0
*Chaetomium anastomosans*	1 (0.6%)	0
*Cladosporium cladosporioides* complex	3 (1.8%)	1 (1.4%)
*Coprinellus domesticus*	1 (0.6%)	0
*Epicoccum mezzettii*	1 (0.6%)	0
***Fusarium***	**80 (47.3%)**	**55 (75.3%)**
**FDSC**	**1 (0.6%)**	**4 (5.5%)**
*Fusarium dimerum*	0	4 (5.5%)
*Fusarium* sp.	1 (0.6%)	0
**FFSC**	**12 (7.1%)**	**5 (6.8%)**
*Fusarium lactis*	1 (0.6%)	0
*Fusarium musae*	2 (1.2%)	1 (1.4%)
*Fusarium proliferatum*	7 (4.1%)	3 (4.1%)
*Fusarium sacchari*	1 (0.6%)	0
*Fusarium verticillioides*	1 (0.6%)	1 (1.4%)
**FIESC**	**1 (0.6%)**	**0**
*Fusarium equiseti*	1 (0.6%)	0
**FOSC**	**10 (5.9%)**	**33 (45.2%)**
**FRSC**	**0**	**1 (1.4%)**
*Fusarium redolens*	0	1 (1.4%)
**FSSC**	**56 (33.1%)**	**12 (16.4%)**
*Fusarium bostrycoides* (FSSC 25 + 35)	3 (1.8%)	0
*Fusarium cyanescens* (FSSC 27)	1 (0.6%)	0
*Fusarium falciforme* (FSSC 3 + 4)	5 (3.0%)	0
*Fusarium ferrugineum* (FSSC 28)	0	1 (1.4%)
*Fusarium keratoplasticum* (FSSC 2)	13 (7.7%)	1 (1.4%)
*Fusarium petroliphilum* (FSSC 1)	20 (11.8%)	7 (9.6%)
*Fusarium metavorans* (FSSC 6)	2 (1.2%)	0
*Fusarium pisi* (FSSC 11)	0	1 (1.4%)
*Fusarium solani* (FSSC 5)	11 (6.5%)	3 (4.1%)
*Fusarium stercicola* (FSSC 44)	0	1 (1.4%)
*Fusarium tonkinense* (FSSC 9)	1 (0.6%)	1 (1.4%)
*Gnomoniopsis idaeicola*	0	1 (1.4%)
***Lecanicillium***	**2 (1.2%)**	**2 (2.7%)**
*Lecanicillium attenuatum*	0	1 (1.4%)
*Lecanicillium coprophilum*	2 (1.2%)	1 (1.4%)
*Lecythophora hoffmannii*	1 (0.6%)	0
*Lichtheimia corymbifera*	1 (0.6%)	0
*Lomentospora prolificans*	2 (1.2%)	0
*Montagnula opulenta*	0	1 (1.4%)
***Penicillium***	**3 (1.8%)**	**2 (2.7%)**
*Penicillium chrysogenum*	0	1 (1.4%)
*Penicillium citrinum*	1 (0.6%)	0
*Penicillium crustosum*	1 (0.6%)	0
*Penicillium rubens*	1 (0.6%)	1 (1.4%)
*Peniophora lycii*	1 (0.6%)	0
*Peroneutypa scoparia*	1 (0.6%)	0
*Petriella setifera*	1 (0.6%)	0
***Plectosphaerella***	**1 (0.6%)**	**1 (1.4%)**
*Plectosphaerella cucumerina*	1 (0.6%)	0
*Plectosphaerella* sp.	0	1 (1.4%)
*Pseudopithomyces* sp.	1 (0.6%)	0
***Purpureocillium***	**21 (12.4%)**	**0**
*Purpureocillium lilacinum*	15 (8.9%)	0
*Purpureocillium sodanum*	6 (3.6%)	0
Rhinocladiella similis	1 (0.6%)	0
***Sarocladium***	**2 (1.2%)**	**2 (2.7%)**
*Sarocladium kiliense*	1 (0.6%)	1 (1.4%)
*Sarocladium spinificis*	1 (0.6%)	0
*Sarocladium strictum*	0	1 (1.4%)
***Scedosporium***	**8 (4.7%)**	**1 (1.4%)**
*Scedosporium apiospermum*	7 (4.1%)	0
*Scedosporium dehoogii*	1 (0.6%)	1 (1.4%)
*Schizophyllum commune*	1 (0.6%)	0
*Scopulariopsis brevicaulis*	1 (0.6%)	0
*Tintelnotia destructans*	2 (1.2%)	1 (1.4%)

**Table 2:** Antifungal susceptibility of species isolated from infected eyes against amphotericin B (AMB), natamycin (NAT), and caspofungin (CAS). For the FSSC, the numbering of the phylogenetic species is given in the first bracket.

Species	MICs in mg/L								
	AMB			NAT			CAS		
	Range	GM	M50/M90	Range	GM	M50/M90	Range	GM	M50/M90
*Alternaria alternata* (10)	0.125–1	0.33	0.25/1	2–4	2.46	2/4	≤0.06–0.5	0.18	0.25/0.25
*Aspergillus flavus* (12)	1–4	1.89	2/4	>32	50.8	>32/>32	0.03–0.25	0.096	0.125/0.25
*Aspergillus fumigatus* (18)	0.125–1	0.31	0.25/1	2–4	2.62	2/4	≤0.06–0.5	0.15	0.125/0.25
*Aspergillus tubingensis* (12)	0.06–0.5	0.14	0.125/0.25	2–4	2.83	2/4	≤0.016–0.5	0.12	0.125/0.5
***Fusarium***									
**FDSC**									
*F. dimerum* (4)	0.5–2	1.19	n.a.	2–4	3.36	n.a.	>8	16	n.a.
*Fusarium* sp. (FDSC) (1)	0.5	n.a.	n.a.	2	n.a.	n.a.	>8	n.a.	n.a.
**FFSC**									
*F. lactis* (1)	1	n.a.	n.a.	4	n.a.	n.a.	8	n.a.	n.a.
*F. musae* (3)	1–4	2	n.a.	2–4	3.17	n.a.	>8	16	n.a.
*F. proliferatum* (10)	1–4	1.62	2/2	4–8	6.96	8/8	8–>8	14.9	>8/>8
*F sacchari* (2)	1–2	1.41	n.a.	4	4	n.a.	>8	16	n.a.
*F. verticillioides* (2)	1–2	1.41	n.a.	4	4	n.a.	>8	16	n.a.
**FIESC**									
*F. equiseti* (1)	2	n.a.	n.a.	4	4	n.a.	0.25	n.a.	n.a.
**F0SC** (43)	0.25–8	1.39	2/2	2–8	4.41	4/8	>8	16	>8/>8
**FRSC**									
*F. redolens* (1)	1	n.a.	n.a.	4	n.a.	n.a.	>8	n.a.	n.a.
**FSSC**									
*F. cyanescens* (FSSC 27) (1)	0.5	n.a.	n.a.	4	n.a.	n.a.	>8	n.a.	n.a.
*F. falciforme* (FSSC 3+4) (5)	1–2	1.15	n.a.	8	n.a.	n.a.	>8	n.a.	n.a.
*F. ferrugineum* (FSSC 28) (1)	1	n.a.	n.a.	4	n.a.	n.a.	>8	n.a.	n.a.
*F. keratoplasticum* (FSSC 2) (14)	1–4	2.56	2/4	4–8	5.12	4/8	>8	16	>8/>8
*F. metavorans* (FSSC 6) (2)	1–8	2.83	n.a.	4–8	5.66	n.a.	8–>8	11.3	n.a.
*F. pisi* (FSSC 11) (1)	2	n.a.	n.a.	8	n.a.	n.a.	>8	n.a.	n.a.
*F. petroliphilum* (FSSC 1) (27)	0.5–8	1.29	2/2	2–8	5.04	4/8	8–>8	15.5	>8/>8
*F. solani* (FSSC 5) (21)	0.5–8	1.39	1/2	4–32	9.59	8/16	>8	16	>8/>8
*F. bostrycoides* (FSSC 25+35) (3)	1	1	n.a.	2–4	2.83	n.a.	>8	16	n.a.
*F. stercicola* (FSSC 44) (1)	2	n.a.	n.a.	4	n.a.	n.a.	>8	n.a.	n.a.
*F. tonkinense* (FSSC 9) (2)	2–4	2.83	n.a.	48	5.66	n.a.	>8	16	n.a.
*Purpureocillium lilacinum* (15)	>16	32	>8/>8	>32	64	>32/>32	0.125–>8	0.87	1/>8
*Purpureocillium sodanum* (7)	>16	32	n.a.	>32	64	n.a.	0.25–>8	0.91	n.a.
*Lomentospora prolificans* (11)	4–>16	26.5	>8/>8	2–8	5.84	8/8	1–8	2.83	4/8
*Scedosporium apiospermum* (17)	1–>16	6.26	8/>8	2–4	2.55	2/4	0.125–2	0.82	1/1
*Scedosporium aurantiacum* (1)	>16	n.a.	n.a.	2	n.a.	n.a.	4	n.a.	n.a.
*Scedosporium dehoogii* (4)	8–>16	13.5	n.a.	2	2	n.a.	1–>8	5.66	n.a.
*Scopulariopsis brevicaulis* (2)	8–>16	16	n.a.	4	4	n.a.	0.5	0.5	n.a.
*Tintelnotia destructans* (3)	0.5–1	0.63	n.a.	1–2	1.41	n.a.	≤0.016–0.25	0.06	n.a.

**Table 3:** Antifungal susceptibility of species isolated from infected eyes against isavuconazole (ISA), itraconazole (ITZ), posaconazole (PCZ), and voriconazole (VCZ). For the FSSC, the numbering of the phylogenetic species is given in the first bracket.

Species	MICs in mg/L											
	ISA			ITZ			PCZ			VCZ		
	Range	GM	M50/M90	Range	GM	M50/M90	Range	GM	M50/M90	Range	GM	M50/M90
*A. alternata* (10)	0.5–16	3.03	4/8	0.25–16	0.93	0.5/1	≤0.016–0.5	0.10	0.125/0.5	0.5–4	1.74	2/2
*A. flavus* (12)	0.5–2	0.75	0.5/2	0.25–2	0.47	0.5/1	0.125–0.5	0.177	0.125/0.5	0.5–1	0.59	0.5/1
*A. fumigatus* (18)	0.125–8	0.71	0.5/8	0.125–>8	0.68	0.5/>8	≤0.016–2	0.06	0.06/0.5	0.25–4	0.58	0.5/2
*A. tubingensis* (12)	1–8	2.92	4/4	1–>8	4	2/>8	0.06–1	0.26	0.25/0.5	0.5–2	1.19	1/2
***Fusarium***												
**FDSC**												
*F. dimerum* (4)	>8	16	n.a.	>8	16	n.a.	>8	16	n.a.	4–8	6.73	n.a.
*Fusarium* sp. (FDSC) (1)	8	n.a.	n.a.	>8	n.a.	n.a.	>8	n.a.	n.a.	4	n.a.	n.a.
**FFSC**												
*F. lactis* (1)	>8	n.a.	n.a.	>8	n.a.	n.a.	>8	n.a.	n.a.	8	n.a.	n.a.
*F. musae* (3)	4	4	n.a.	>8	16	n.a.	1–2	1.26	n.a.	2–4	2.52	n.a.
*F. proliferatum* (10)	>8	16	>8/>8	>8	16	n.a.	4–>8	6.06	>8/>8	4–8	5.66	4/8
*F sacchari* (2)	2–4	2.83	n.a.	>8	11	n.a.	0.5	0.5	n.a.	2	2	n.a.
*F. verticillioides* (2)	2–4	2.83	n.a.	2–>8	5.7	n.a.	0.25–1	0.5	n.a.	1–2	1.41	n.a.
**FIESC**												
*F. equiseti* (1)	2	n.a.	n.a.	1	n.a.	n.a.	0.5	n.a.	n.a.	1	n.a.	n.a.
**F0SC** (43)	4–>8	13.0	>8/>8	>8	16	>8/>8	1–>8	12.77	>8/>8	2–>8	4.78	4/>8
**FRSC**												
*F. redolens* (1)	>8	n.a.	n.a.	>8	n.a.	n.a.	>8	n.a.		4	n.a.	n.a.
**FSSC**												
*F. cyanescens* (FSSC 27) (1)	>8	n.a.	n.a.	>8	n.a.	n.a.	>8	n.a.	n.a.	>8	n.a.	n.a.
*F. falciforme* (FSSC 3+4) (5)	>8	16	n.a.	>8	16	>8/>8	>8	16	n.a.	>8	16	n.a.
*F. ferrugineum* (FSSC 28) (1)	>8	n.a.	n.a.	>8	n.a.	n.a.	>8	n.a.	n.a.	4	n.a.	n.a.
*F. keratoplasticum* (FSSC 2) (14)	>8	16	>8/>8	>8	16	>8/>8	>8	16	>8/>8	4–>8	12.50	>8/>8
*F. metavorans* (FSSC 6) (2)	8–>8	11.3	n.a.	8–>8	11	n.a.	8–>8	11.3	n.a.	4–8	5.66	n.a.
*F. pisi* (FSSC 11) (1)	>8	n.a	n.a.	>8	n.a	n.a.	>8	n.a	n.a.	>8	n.a	n.a.
*F. petroliphilum* (FSSC 1) (27)	>8	16	>8/>8	>8	16	>8/>8	>8	16	>8/>8	4–>8	14.4	>8/>8
*F. solani* (FSSC 5) (21)	>8	16	>8/>8	>8	16	>8/>8	>8	15.5	>8/>8	4–>8	12.7	>8/>8
*F. bostrycoides*(FSSC 25+35) (3)	>8	16	n.a.	>8	16	n.a.	>8	16	n.a.	1–>8	4	n.a.
*F. stercicola* (FSSC 44) (1)	>8	n.a	n.a.	>8	n.a	n.a.	>8	n.a	n.a.	>8	n.a	n.a.
*F. tonkinense* (FSSC 9) (2)	>8	16	n.a.	>8	16	n.a.	>8	16	n.a.	>8	16	n.a.
*P. lilacinum* (15)	0.06–2	0.34	0.5/1	1–>8	5.79	>8/>8	0.06–1	0.16	0.125/0.5	0.125–0.5	0.24	0.25/0.5
*P. sodanum* (7)	0.125–1	0.35	n.a.	2–>8	###	n.a.	0.06–0.5	0.15	n.a.	0.125–0.5	0.25	n.a.
*L. prolificans* (11)	4–>8	12.40	>8/>8	>8	16	>8/>8	>8	16	>8/>8	4–>8	13.2	>8/>8
*S. apiospermum* (17)	>8	11.60	>8/>8	2–8	12	n.a.	1–>8	5.77	4/>8	0.5–1	0.75	1/1
*S. aurantiacum* (1)	8	n.a	n.a.	>8	n.a	n.a.	2	n.a	n.a	0.5	n.a	n.a
*S. dehoogii* (4)	2–8	4	n.a.	1–>8	2	n.a.	0.25–2	0.71	n.a	0.25–0.5	0.42	n.a
*Scopulariopsis brevicaulis* (2)	>8	16.00	n.a.	>8	16	n.a.	>8	16	n.a	>8	16	n.a
*Tintelnotia destructans* (3)	4–8	6.35	n.a.	0.25–0.5	0.40	n.a.	0.06–0.125	0.10	n.a	0.5–1	0.79	n.a

## Data Availability

All raw data can be found in Table S1.

## Supplementary Materials

The following are available online at https://www.mdpi.com/article/10.3390/jof7070511/s1, Table S1: Strains studied. their strain numbers. sources. GenBank accession numbers. and antifungal susceptibilities for amphotericin B (AMB). natamycin (NAT). isavuconazole (ISA). itraconazole (ITZ). posaconazole (PCZ). voriconazole (VCZ) and caspofungin (CAS). Strains from the same patient are marked. Table S2: Markers amplified for the identification of eye infecting filamentous fungi, primers and annealing temperatures used for PCR.
