# Supplementary material for: Eye Infections Caused by Filamentous Fungi: Spectrum and Antifungal Susceptibility of the Prevailing Agents in Germany
Source: J Fungi (Basel). 2021 Jun 26;7(7):511. doi: 10.3390/jof7070511 (PMC8307352; doi:10.3390/jof7070511)
Supplement: Supplementary file 1 [file jof-07-00511-s001.zip › Walther et al._table S1 rev.pdf]

Table S1: Strains studied. their strain numbers. sources. GenBank accession numbers. and antifungal susceptibilities for amphotericin B (AMB). natamycin (NAT). isavuconazole (ISA). itraconazole (ITZ). posaconazole (PCZ). voriconazole (VCZ) and caspofungin (CAS). Strains from the same patient are marked.

Strain number	Species	Source	Strains from one patient	GenBank nr.	AMB	NAT	ISA	ITZ	PCZ	VCZ
JMRC:NRZ:0637	*Alternaria alternata*	Eye		MZ359395	0.25	2	4	1	0.06	2
JMRC:NRZ:1653	*Alternaria alternata*	Cornea		MZ359397	0.25	2	4	0.5	0.125	2
JMRC:NRZ:2361	*Alternaria alternata*	Corneal swab		MZ359396	0.125	2	4	2	0.5	2
JMRC:NRZ:2613	*Alternaria alternata*	[Wound. tibia]		MZ359398	1	4	1	1	0.25	2
JMRC:NRZ:2899	*Alternaria alternata*	Corneal swab		MZ359400	1	4	1	0.5	0.06	1
JMRC:NRZ:0363	*Alternaria alternata*	Corneal swab		MZ359406	0.5		8	0.5	0.125	2
JMRC:NRZ:1133	*Alternaria alternata*	[Skin graft]		MZ359402	0.25	2	4	1	0.125	2
JMRC:NRZ:1137	*Alternaria alternata*	Corneal swab		MZ359401	0.25	2	4	0.5	≤0.016	2
JMRC:NRZ:1261	*Alternaria alternata*	Swab of cornea and conjunctiva		MZ359404	0.125	4	>8	>8	0.25	2
JMRC:NRZ:2180	*Alternaria alternata*	[Sinus (chronic Pansinusitis)]		MZ359399	0.5	2	8	0.5	0.125	4
JMRC:NRZ:2231	*Alternaria alternata*	[Nasal lavage]		MZ359403	0.5	2	0.5	0.25	0.03	0.5
JMRC:NRZ:2835	*Alternaria alternata*	Corneal ulcer		MZ359405						
JMRC:NRZ:2912	*Alternaria hordeicola*	Contact lens cleaning solution	patient D	MZ359407						
JMRC:NRZ:0602	*Alternaria rosae*	Contact lens (Corneal ulcer)		MZ359408						
JMRC:NRZ:2646	*Arthrographis kalrae*	Cornea		MZ382438						
JMRC:NRZ:2792	*Arthrographis kalrae*	Corneal scraping		MZ382442						
JMRC:NRZ:1574	*Aspergillus cibarius*	Cornea		MZ382424						
JMRC:NRZ:0634	*Aspergillus flavus*	Tissue of the eye. intraoperative		MZ359540	4	32	0.5	0.25	0.125	0.5
JMRC:NRZ:0980	*Aspergillus flavus*	Swab of the eye		MZ359541	4	>32	2	2	0.5	1
JMRC:NRZ:1469	*Aspergillus flavus*	Corneal swab		MZ359543	2	32	0.5	0.5	0.5	0.5
JMRC:NRZ:1656	*Aspergillus flavus*	Swab of conjunctiva		MZ359544	1	32	0.5	0.5	0.125	0.5
JMRC:NRZ:1758	*Aspergillus flavus*	Contact lens (Keratitis)		MZ359545	1	>32	0.5	0.25	0.125	1
JMRC:NRZ:2373	*Aspergillus flavus*	[Bronchial aspirate]		MZ359546	2	>32	2	1	0.25	1
JMRC:NRZ:2671	*Aspergillus flavus*	[Tracheal secretion]		MZ359547	2	>32	0.5	0.5	0.125	0.5
JMRC:NRZ:2841	*Aspergillus flavus*	[Tracheal secretion]		MZ359548	1	>32	0.5	0.25	0.125	0.5
JMRC:NRZ:2856	*Aspergillus flavus*	[Peritoneal fluid]		MZ359549	2	>32	1	0.25	0.125	0.5
JMRC:NRZ:3010	*Aspergillus flavus*	[Swab of ear canal]		MZ359550	2	32	1	0.5	0.125	0.5
JMRC:NRZ:0055	*Aspergillus flavus*	Corneal ulcer		MZ359539	4	>32	1	1	0.25	0.5
JMRC:NRZ:1374	*Aspergillus flavus*	Contact lens (Keratitis)		MZ359542	1	>32	0.5	0.25	0.125	0.5
JMRC:NRZ:0238	*Aspergillus fumigatus*	Swab of cornea		MZ359583	0.25	4	0.5	1	0.06	0.5
JMRC:NRZ:0404	*Aspergillus fumigatus*	Vitreous body		MZ359585	0.25	2	0.5	0.5	0.03	0.5
JMRC:NRZ:0635	*Aspergillus fumigatus*	Corneal scraping		MZ359584	0.5	2	1	0.5	0.125	0.5

JMRC:NRZ:0932	*Aspergillus fumigatus*	Contact lens (Keratitis)	patient B	MZ359586	0.25	2	0.5	0.5	0.016	0.5
JMRC:NRZ:1301	*Aspergillus fumigatus*	Swab of cornea and conjunctiva		MZ359629	1	4	0.5	2	0.125	0.25
JMRC:NRZ:1343	*Aspergillus fumigatus*	Corneal ulcer		MZ359628	1	2	0.5	1	0.125	0.5
JMRC:NRZ:1919	*Aspergillus fumigatus*	cornea		MZ359630	1	2	0.125	0.25	≤0.015	0.25
JMRC:NRZ:2134	*Aspergillus fumigatus*	Swab of cornea		MZ359631	0.125	2	0.5	0.25	0.03	0.5
JMRC:NRZ:2222	*Aspergillus fumigatus*	Corneal scraping		MZ359587	0.125	2	0.5	0.125	≤0.015	0.5
JMRC:NRZ:2493	*Aspergillus fumigatus*	Swab of corneal ulcer		MZ359632	0.25	4	0.5	0.5	0.03	0.5
JMRC:NRZ:2602	*Aspergillus fumigatus*	[Tissue of the nasal cavity]		MZ359633	0.125	4	1	0.25	≤0.016	0.5
JMRC:NRZ:2697	*Aspergillus fumigatus*	Cornea		MZ359635	0.125	2	8	>8	0.5	4
JMRC:NRZ:2875	*Aspergillus fumigatus*	[Sputum]		MZ359634	0.5	2	8	>8	2	2
JMRC:NRZ:2987	*Aspergillus fumigatus*	[Swab of ear]		MZ359588	0.5	4	0.5	0.25	≤0.016	0.5
JMRC:NRZ:3018	*Aspergillus fumigatus*	[Bronchial alveolar larvage]		MZ359589	0.25	2	0.5	0.5	0.06	0.5
JMRC:NRZ:3019	*Aspergillus fumigatus*	[Bronchial secretion]		MZ359590	0.5	2	0.5	0.5	0.06	0.5
JMRC:NRZ:3042	*Aspergillus fumigatus*	[Palacos endoprothesis]		MZ359591	0.125	4	1	0.5	0.03	0.5
JMRC:NRZ:2674	*Aspergillus hiratsukae*	Corneal swab	patient C	MZ382439						
JMRC:NRZ:2418	*Aspergillus sydowii*	Transport medium of corneal graft		MZ382444						
JMRC:NRZ:0484	*Aspergillus terreus*	Vitreous body		MZ382421						
JMRC:NRZ:0060	*Aspergillus tubingensis*	Vitreous body		MZ359602	0.06	2	n.a.	4	0.12	1
JMRC:NRZ:1858	*Aspergillus tubingensis*	[Ear canal]		MZ359593	0.125	4	4	8	0.5	2
JMRC:NRZ:2505	*Aspergillus tubingensis*	[Tracheal secretion]		MZ359601	0.25	4	4	2	0.5	1
JMRC:NRZ:2713	*Aspergillus tubingensis*	[Ear canal]		MZ359600	0.125	4	4	2	0.25	2
JMRC:NRZ:0933	*Aspergillus tubingensis*	Contact lens (keratitis)	patient B	MZ359592	0.06	4	2	>8	0.06	1
JMRC:NRZ:1323	*Aspergillus tubingensis*	[Ear canal]		MZ359596	0.125	4	1	1	0.25	0.5
JMRC:NRZ:1808	*Aspergillus tubingensis*	[Ear canal]		MZ359597	0.25	2	2	2	0.5	1
JMRC:NRZ:2096	*Aspergillus tubingensis*	[Ear canal]		MZ359599	0.5	2	2	2	0.5	1
JMRC:NRZ:2116	*Aspergillus tubingensis*	[Sputum]		MZ359594	0.125	2	4	1	0.25	1
JMRC:NRZ:2131	*Aspergillus tubingensis*	Swap of cornea		MZ359598	0.125	4	2	>8	0.125	1
JMRC:NRZ:2263	*Aspergillus tubingensis*	[Ear canal]		MZ359595	0.06	2	4	8	0.125	2
JMRC:NRZ:2396	*Aspergillus tubingensis*	[Swap of ear]		MZ359603	0.25	2	8	>8	1	2
JMRC:NRZ:2692	*Aspergillus udagawae*	Cornea		MZ359636						
JMRC:NRZ:2751	*Aureobasidium pullulans*	Anterior chamber		MZ382441						
JMRC:NRZ:2647	*Chaetomium anastomosans*	Swab of eye		MZ382423						
JMRC:NRZ:0420	*Cladosporium cladosporioides* complex	Transport medium of cornal graft		MZ359612	0.5	0.5	2	0.5	0.03	1
JMRC:NRZ:0867	*Cladosporium cladosporioides* complex	[Bronchial alveolar larvage]		MZ359613	1	0.5	8	1	0.25	2
JMRC:NRZ:2108	*Cladosporium cladosporioides* complex	Swap of conjunctiva		MZ359610	0.25	1	>8	2	0.5	>8
JMRC:NRZ:2183	*Cladosporium cladosporioides* complex	Swap of conjunctiva		MZ359609	0.25	1	1	≤0.016	≤0.016	1
JMRC:NRZ:2194	*Cladosporium cladosporioides* complex	[Skin]		MZ359608	1	0.5	>8	2	1	>8
JMRC:NRZ:2790	*Cladosporium cladosporioides* complex	Intraoperative swap of eye with corneal		MZ359611	0.5	1	>8	2	0.5	>8

		opacity								
JMRC:NRZ:2364	*Coprinellus domesticus* (syn. *Hormographiella verticillata)*	Tissue of the eye		MZ382437						
JMRC:NRZ:1612	*Epicoccum mezzettii*	Corneal scraping		MZ382422						
JMRC:NRZ:0061	*Fusarium bostrycoides* (FSSC 25 and 35)	Eye		MF467477	1	2	n.a.	>8	>8	1
JMRC:NRZ:0483	*Fusarium bostrycoides* (FSSC 25 and 35)	Cornea (keratouveitis)		MZ359425	1	4	>8	>8	>8	>8
JMRC:NRZ:1427	*Fusarium bostrycoides* (FSSC 25 and 35)	Anterior chamber	patient A	MZ359482	1	4	>8	>8	>8	4
JMRC:NRZ:0639	*Fusarium cyanescens* (FSSC 27)	Corneal scraping		MZ359432	0.5	4	>8	>8	>8	>8
JMRC:NRZ:1275	*Fusarium dimerum*	n.a. (Contact lens associated keratitis)		MZ359477	2	4	>8	>8	>8	4
JMRC:NRZ:1581	*Fusarium dimerum*	Contact lens		MZ359492	1	4	>8	>8	>8	8
JMRC:NRZ:2879	*Fusarium dimerum*	Contact lens cleaning solution		MZ359533	0.5	2	>8	>8	>8	8
JMRC:NRZ:2397	*Fusarium dimerum*	Contact lens (keratitis)		MZ359527	2	4	>8	>8	>8	8
JMRC:NRZ:1646	*Fusarium equiseti*	Corneal tissue		MZ382432	2	4	2	1	0.5	1
JMRC:NRZ:0138	*Fusarium falciforme*	Eye (Keratitis)		MF467462	2	8	n.a.	>8	>8	>8
JMRC:NRZ:0369	*Fusarium falciforme*	Swap of conjunctiva		MZ359417	1	8	>8	>8	>8	>8
JMRC:NRZ:1732	*Fusarium falciforme*	Cormeal swab		MZ359500	1	8	>8	>8	>8	>8
JMRC:NRZ:2181	*Fusarium falciforme*	Cormeal swab		MZ359520	0.5	8	>8	>8	>8	>8
JMRC:NRZ:2853	*Fusarium falciforme*	Corneal scraping		MZ359532	1	8	>8	>8	>8	>8
JMRC:NRZ:2179	*Fusarium ferrugineum (FSSC 28)*	Contact lens (keratitis)		MZ359519	1	4	>8	>8	>8	4
JMRC:NRZ:0049	*Fusarium keratoplasticum*	Swap of eye		MF467485	4	4	>8	>8	>8	>8
JMRC:NRZ:0131	*Fusarium keratoplasticum*	Corneal swap		MF467461	4	8	n.a.	>8	>8	8
JMRC:NRZ:0364	*Fusarium keratoplasticum*	Corneal scraping		MZ359415	2	4	>8	>8	>8	>8
JMRC:NRZ:0366	*Fusarium keratoplasticum*	Corneal swap		MZ359416	1	8	>8	>8	>8	>8
JMRC:NRZ:0540	*Fusarium keratoplasticum*	(Keratitis)		MZ359427	4	4	>8	>8	>8	>8
JMRC:NRZ:1078	*Fusarium keratoplasticum*	Corneal scraping		MZ359458	2	4	>8	>8	>8	4
JMRC:NRZ:1162	*Fusarium keratoplasticum*	Corneal scraping		MZ359469	2	4	>8	>8	>8	>8
JMRC:NRZ:1188	*Fusarium keratoplasticum*	Cornea		MZ359471	2	4	>8	>8	>8	>8
JMRC:NRZ:1273	*Fusarium keratoplasticum*	Corneal swap		MZ359476	4	8	>8	>8	>8	8
JMRC:NRZ:1511	*Fusarium keratoplasticum*	Contact lens (keratitis)		MZ359486	2	8	>8	>8	>8	>8
JMRC:NRZ:1683	*Fusarium keratoplasticum*	(Keratitis)		MZ359498	2	4	>8	>8	>8	>8
JMRC:NRZ:1983	*Fusarium keratoplasticum*	(Keratitis)		MZ359511	4	8	>8	>8	>8	>8
JMRC:NRZ:2448	*Fusarium keratoplasticum*	Corneal swap		MZ359528	4	4	>8	>8	>8	8
JMRC:NRZ:2093	*Fusarium keratoplasticum*	Corneal swap		MZ359517	2	4	>8	>8	>8	>8
JMRC:NRZ:0506	*Fusarium lactis*	Corneal scraping		MZ359426	1	4	>8	>8	>8	8
JMRC:NRZ:0892	*Fusarium metavorans*	Corneal scraping		MZ359447	8	8	>8	>8	>8	8
JMRC:NRZ:1571	*Fusarium metavorans*	Corneal scraping		MZ359491	1	4	8	8	8	4
JMRC:NRZ:0627	*Fusarium musae*	Corneal swap		MZ359431	1	2	4	>8	1	2
JMRC:NRZ:1505	*Fusarium musae*	Contact lens		MZ359485	4	4	4	>8	2	2

JMRC:NRZ:0576	*Fusarium musae*	Corneal scraping		MZ359430	2	4	4	>8	1	4
JMRC:NRZ:0027	*Fusarium oxysporum* complex	Contact lens (keratitis)		MF467452	2	4	>8	>8	>8	4
JMRC:NRZ:0189	*Fusarium oxysporum* complex	Contact lens (corneal ulcer)		MF467453	2	4	>8	>8	>8	4
JMRC:NRZ:0198	*Fusarium oxysporum* complex	Contact lens cleaning solution (corneal ulcer)		MF467455	1	4	>8	>8	>8	4
JMRC:NRZ:0204	*Fusarium oxysporum* complex	Contact lens case (keratitis)		MF467456	2	4	>8	>8	>8	4
JMRC:NRZ:0260	*Fusarium oxysporum* complex	Contact lens cleaning solution (keratitis)		MF467454	0.5	4	8	>8	>8	4
JMRC:NRZ:0304	*Fusarium oxysporum* complex	Corneal swab		MZ359412	0.5	4	>8	>8	>8	4
JMRC:NRZ:0408	*Fusarium oxysporum* complex	Contact lens cleaning solution (keratitis)		MZ359419	2	4	>8	>8	>8	4
JMRC:NRZ:0412	*Fusarium oxysporum* complex	Corneal swab		MZ359421	1	8	8	>8	>8	8
JMRC:NRZ:0463	*Fusarium oxysporum* complex	Contact lens (keratitis)		MZ359422	2	4	>8	>8	>8	>8
JMRC:NRZ:0477	*Fusarium oxysporum* complex	Swab of eye		MZ359423	2	4	>8	>8	4	4
JMRC:NRZ:0646	*Fusarium oxysporum* complex	Contact lens cleaning solution (keratitis)		MZ359433	0.5	2	>8	>8	>8	4
JMRC:NRZ:0648	*Fusarium oxysporum* complex	Contact lens cleaning solution		MZ359434	0.25	4	8	>8	>8	4
JMRC:NRZ:0701	*Fusarium oxysporum* complex	Contact lens (keratitis)		MZ359437	1	8	>8	>8	>8	8
JMRC:NRZ:0726	*Fusarium oxysporum* complex	Contact lens (corneal ulcer)		MZ359439	1	4	>8	>8	>8	4
JMRC:NRZ:0781	*Fusarium oxysporum* complex	(Keratitis)		MZ359443	2	4	>8	>8	>8	8
JMRC:NRZ:0784	*Fusarium oxysporum* complex	Contact lens (corneal infiltrates)		MZ359444	1	4	>8	>8	>8	8
JMRC:NRZ:0888	*Fusarium oxysporum* complex	Contact lens		MZ359446	1	4	>8	>8	>8	4
JMRC:NRZ:0940	*Fusarium oxysporum* complex	Contact lens case (keratitis)		MZ359448	1	4	>8	>8	>8	>8
JMRC:NRZ:0945	*Fusarium oxysporum* complex	Contact lens (keratitis)		MZ359449	1	4	>8	>8	>8	4
JMRC:NRZ:1014	*Fusarium oxysporum* complex	Contact lens		MZ359451	4	4	>8	>8	>8	4
JMRC:NRZ:1051	*Fusarium oxysporum* complex	Contact lens (keratitis)		MZ359454	2	4	8	>8	8	2
JMRC:NRZ:1055	*Fusarium oxysporum* complex	Contact lens		MZ359461	2	4	>8	>8	>8	4
JMRC:NRZ:1071	*Fusarium oxysporum* complex	Contact lens (keratitis)		MZ359457	0.5	4	8	>8	>8	2
JMRC:NRZ:1085	*Fusarium oxysporum* complex	Contact lens (keratitis)		MZ359459	2	4	8	>8	8	2
JMRC:NRZ:1098	*Fusarium oxysporum* complex	Contact lens (keratitis)		MZ359462	2	4	>8	>8	>8	4
JMRC:NRZ:1124	*Fusarium oxysporum* complex	Contact lens (keratitis)		MZ359465	1	4	>8	>8	>8	4
JMRC:NRZ:1204	*Fusarium oxysporum* complex	Contact lens (keratitis)		MZ359473	2	4	>8	>8	>8	>8
JMRC:NRZ:1220	*Fusarium oxysporum* complex	Corneal scraping		MZ359474	2	4	8	>8	1	2
JMRC:NRZ:1300	*Fusarium oxysporum* complex	(Keratitis)		MZ359478	2	4	8	>8	>8	2
JMRC:NRZ:1460	*Fusarium oxysporum* complex	Contact lens (keratitis)		MZ359484	2	8	>8	>8	>8	4
JMRC:NRZ:1662	*Fusarium oxysporum* complex	Corneal swab		MZ359496	0.5	8	>8	>8	>8	8
JMRC:NRZ:1665	*Fusarium oxysporum* complex	Contact lens (keratitis)		MZ359497	2	4	>8	>8	>8	4
JMRC:NRZ:1698	*Fusarium oxysporum* complex	Contact lens cleaning solution (keratitis)		MZ359499	1	4	>8	>8	>8	4
JMRC:NRZ:1768	*Fusarium oxysporum* complex	Corneal swab		MZ359501	2	8	8	>8	>8	4
JMRC:NRZ:1817	*Fusarium oxysporum* complex	Contact lens cleaning solution (keratitis)	patient F	MZ359502	2	8	>8	>8	>8	8
JMRC:NRZ:1912	*Fusarium oxysporum* complex	Eye swab		MZ359507	1	4	>8	>8	>8	>8

JMRC:NRZ:2053	*Fusarium oxysporum* complex	Contact lens (keratitis)		MZ359515	8	4	4	>8	2	2
JMRC:NRZ:2065	*Fusarium oxysporum* complex	Contact lens case (keratitis)		MZ359516	2	4	8	>8	2	2
JMRC:NRZ:2235	*Fusarium oxysporum* complex	Corneal scraping		MZ359522	1	4	>8	>8	>8	>8
JMRC:NRZ:2304	*Fusarium oxysporum* complex	Contact lens (keratitis)		MZ359523	2	8	>8	>8	>8	>8
JMRC:NRZ:2316	*Fusarium oxysporum* complex	Contact lens (keratitis)		MZ359524	2	4	>8	>8	>8	4
JMRC:NRZ:2700	*Fusarium oxysporum* complex	Contact lens (keratitis)		MZ359531	1	4	>8	>8	>8	4
JMRC:NRZ:0012	*Fusarium petroliphilum*	Eye		MF467470	2	4	>8	>8	>8	>8
JMRC:NRZ:0017	*Fusarium petroliphilum*	Anterior chamber		MF467472	1	4	>8	>8	>8	>8
JMRC:NRZ:0059	*Fusarium petroliphilum*	Corneal swab		MF467474	2	4	n.a.	>8	>8	4
JMRC:NRZ:0086	*Fusarium petroliphilum*	Corneal scraping		MF467468	1	4	n.a.	>8	>8	>8
JMRC:NRZ:0106	*Fusarium petroliphilum*	Corneal swab		MF467465	0.5	8	n.a.	>8	>8	>8
JMRC:NRZ:0278	*Fusarium petroliphilum*	Eye		MZ359411	0.5	4	>8	>8	>8	>8
JMRC:NRZ:0311	*Fusarium petroliphilum*	Conjunctival swab		MZ359413	1	8	>8	>8	>8	>8
JMRC:NRZ:0337	*Fusarium petroliphilum*	Corneal swab		MZ359414	2	4	>8	>8	>8	8
JMRC:NRZ:0411	*Fusarium petroliphilum*	Anterior chamber		MZ359420	2	8	>8	>8	>8	>8
JMRC:NRZ:0480	*Fusarium petroliphilum*	Cornea		MZ359424	1	4	>8	>8	>8	>8
JMRC:NRZ:0559	*Fusarium petroliphilum*	Cornea		MZ359428	4	8	>8	>8	>8	>8
JMRC:NRZ:0574	*Fusarium petroliphilum*	Contact lens		MZ359429	1	4	>8	>8	>8	>8
JMRC:NRZ:0710	*Fusarium petroliphilum*	Corneal scraping		MZ359438	1	2	>8	>8	>8	>8
JMRC:NRZ:0757	*Fusarium petroliphilum*	Contact lens		MZ359440	0.5	8	>8	>8	>8	>8
JMRC:NRZ:0825	*Fusarium petroliphilum*	Contact lens		MZ359445	0.5	8	>8	>8	>8	>8
JMRC:NRZ:1005	*Fusarium petroliphilum*	Corneal scraping		MZ359450	8	4	>8	>8	>8	>8
JMRC:NRZ:1039	*Fusarium petroliphilum*	Vitreous body		MZ359452	1	4	>8	>8	>8	>8
JMRC:NRZ:1066	*Fusarium petroliphilum*	Contact lens (keratitis)		MZ359455	2	4	>8	>8	>8	>8
JMRC:NRZ:1097	*Fusarium petroliphilum*	Contact lens (keratitis)		MZ359460	2	4	>8	>8	>8	>8
JMRC:NRZ:1145	*Fusarium petroliphilum*	Contact lens (keratitis)		MZ359466	4	4	>8	>8	>8	>8
JMRC:NRZ:1908	*Fusarium petroliphilum*	Corneal swab		MZ359506	1	8	>8	>8	>8	>8
JMRC:NRZ:1921	*Fusarium petroliphilum*	Corneal scraping		MZ359508	1	8	>8	>8	>8	>8
JMRC:NRZ:2225	*Fusarium petroliphilum*	Contact lens (blepharokonjunktivitis)	patient G	MZ359521	1	8	>8	>8	>8	>8
JMRC:NRZ:1118	*Fusarium petroliphilum*	Contact lens (keratitis)		MZ359464	2	4	>8	>8	>8	>8
JMRC:NRZ:1186	*Fusarium petroliphilum*	(Keratitis)		MZ359470	2	8	>8	>8	>8	>8
JMRC:NRZ:2490	*Fusarium petroliphilum*	Anterior chamber		MZ359529	0.5	4	>8	>8	>8	>8
JMRC:NRZ:2888	*Fusarium petroliphilum*	Corneal swab		MZ359534	1	4	>8	>8	>8	8
JMRC:NRZ:1818	*Fusarium pisi* (FSSC 11)	Contact lens cleaning solution (keratitis)	patient F	MZ359503	2	8	>8	>8	>8	>8
JMRC:NRZ:0196	*Fusarium proliferatum*	Contact lens (keratitis)		MZ359409	2	8	>8	>8	8	4
JMRC:NRZ:0202	*Fusarium proliferatum*	Anterior chamber		MZ359410	1	8	>8	>8	>8	8
JMRC:NRZ:0379	*Fusarium proliferatum*	(Keratitis)		MZ359418	2	8	>8	>8	>8	8
JMRC:NRZ:0657	*Fusarium proliferatum*	Corneal scraping		MZ359435	1	**8**	>8	>8	>8	8

JMRC:NRZ:0773	*Fusarium proliferatum*	Cornea		MZ359441	1	4	>8	>8	>8	4
JMRC:NRZ:1099	*Fusarium proliferatum*	Contact lens (keratitis)		MZ359463	2	8	>8	>8	4	4
JMRC:NRZ:1317	*Fusarium proliferatum*	Corneal swab		MZ359479	1	4	>8	>8	>8	4
JMRC:NRZ:1563	*Fusarium proliferatum*	Corneal scraping		MZ359488	4	8	>8	>8	>8	4
JMRC:NRZ:1607	*Fusarium proliferatum*	Contact lens cleaning solution		MZ359493	2	8	>8	>8	>8	8
JMRC:NRZ:2932	*Fusarium proliferatum*	Eye swab		MZ359536	4	8	>8	>8	>8	8
JMRC:NRZ:2349	*Fusarium redolens*	Contact lens (keratitis)		MZ359525	1	4	>8	>8	>8	4
JMRC:NRZ:1394	*Fusarium sacchari*	Eye swab		MZ359481	1	4	2	8	0.5	2
JMRC:NRZ:2042	*Fusarium sacchari*	[Blood]		MZ359514	2	4	4	>8	0.5	2
JMRC:NRZ:0205	*Fusarium solani*	Vitreous body		MF467463	2	8	>8	>8	>8	>8
JMRC:NRZ:1068	*Fusarium solani*	Contact lens (keratitis)		MZ359456	1	8	>8	>8	>8	8
JMRC:NRZ:1154	*Fusarium solani*	Eye swab		MZ359468	1	8	>8	>8	>8	>8
JMRC:NRZ:1243	*Fusarium solani*	Corneal swab		MZ359475	2	8	>8	>8	>8	4
JMRC:NRZ:1329	*Fusarium solani*	[Nasal mucosa]		MZ359480	2	16	>8	>8	>8	8
JMRC:NRZ:1445	*Fusarium solani*	[Wound. foot]		MZ359483	0.5	32	>8	>8	>8	>8
JMRC:NRZ:1515	*Fusarium solani*	Cornea		MZ359487	2	8	>8	>8	>8	>8
JMRC:NRZ:1566	*Fusarium solani*	Contact lens (keratitis)		MZ359490	1	4	>8	>8	>8	4
JMRC:NRZ:1655	*Fusarium solani*	(Keratitis)		MZ359495	1	8	>8	>8	>8	>8
JMRC:NRZ:1864	*Fusarium solani*	Corneal scraping		MZ359504	2	16	>8	>8	>8	>8
JMRC:NRZ:1876	*Fusarium solani*	[Wound. tight]		MZ359505	1	8	>8	>8	>8	>8
JMRC:NRZ:1939	*Fusarium solani*	[Wound. arm]		MZ359509	0.5	16	>8	>8	8	>8
JMRC:NRZ:2155	*Fusarium solani*	[Wound. finger]		MZ359518	8	8	>8	>8	>8	>8
JMRC:NRZ:2654	*Fusarium solani*	[Wound. lower leg]		MZ359530	2	32	>8	>8	>8	>8
JMRC:NRZ:2964	*Fusarium solani*	unknown		MZ359537	1	16	>8	>8	>8	>8
JMRC:NRZ:2969	*Fusarium solani*	Anterior chamber		MZ359538	2	16	>8	>8	>8	>8
JMRC:NRZ:0658	*Fusarium solani*	Corneal scraping		MZ359436	1	4	>8	>8	>8	>8
JMRC:NRZ:1962	*Fusarium solani*	Corneal scraping		MZ359510	1	8	>8	>8	>8	>8
JMRC:NRZ:2030	*Fusarium solani*	Cornea		MZ359512	2	8	>8	>8	>8	>8
JMRC:NRZ:2031	*Fusarium solani*	Contact lens (keratitis)		MZ359513	1	8	>8	>8	>8	8
JMRC:NRZ:2910	*Fusarium solani*	Cornea		MZ359535	2	16	>8	>8	>8	>8
JMRC:NRZ:0776	*Fusarium sp.* (FDSC)	Eye		MZ359442	0.5	2	8	>8	>8	4
JMRC:NRZ:1198	*Fusarium stercicola* (FSSC 44)	Contact lens (keratitis)		MZ359472	2	4	>8	>8	>8	>8
JMRC:NRZ:0233	*Fusarium tonkinense* (FSSC 9)	(Keratitis/endophthalmitis)		MF467464	2	4	>8	>8	>8	>8
JMRC:NRZ:1050	*Fusarium tonkinense* (FSSC 9)	(Keratitis)		MZ359453	4	8	>8	>8	>8	>8
JMRC:NRZ:1650	*Fusarium verticillioides*	Contact lens (keratitis)		MZ359494	1	4	2	2	0.25	1
JMRC:NRZ:2354	*Fusarium verticillioides*	Anterior chamber		MZ359526	2	4	4	>8	1	2
JMRC:NRZ:1244	*Gnomoniopsis idaeicola*	Eye with foreign body		MZ382428						
JMRC:NRZ:1289	*Lecanicillium attenuatum*	Contact lens cleaning solution (keratitis)		MZ359614						

JMRC:NRZ:1201	*Lecanicillium coprophilum*	Corneal swab		MZ359616						
JMRC:NRZ:2210	*Lecanicillium coprophilum*	Contact lens (blepharokonjunktivitis)	patient G	MZ359615						
JMRC:NRZ:2358	*Lecythophora hoffmannii*	Corneal swab		MZ382436						
JMRC:NRZ:0394	*Lichtheimia corymbifera*	(Keratitis)		MZ382426						
JMRC:NRZ:0598	*Lomentospora prolificans*	Contact lens (keratitis)		MZ359557	>16	4	>8	>8	>8	>8
JMRC:NRZ:1636	*Lomentospora prolificans*	[Blood]		MZ359553	4	2	4	>8	>8	4
JMRC:NRZ:2154	*Lomentospora prolificans*	[Wound. finger]		MZ359551	>16	8	8	>8	>8	8
JMRC:NRZ:2188	*Lomentospora prolificans*	[Blood]		MZ359560	>16	8	>8	>8	>8	>8
JMRC:NRZ:2335	*Lomentospora prolificans*	[Sputum]		MZ359556	>16	8	>8	>8	>8	>8
JMRC:NRZ:2688	*Lomentospora prolificans*	[Wound. hand]		MZ359552	>16	8	>8	>8	>8	>8
JMRC:NRZ:1001	*Lomentospora prolificans*	Corneal swab		MZ359559	>16	4	>8	>8	>8	>8
JMRC:NRZ:1061	*Lomentospora prolificans*	[Blood]		MZ359558	>16	8	8	>8	>8	>8
JMRC:NRZ:2083	*Lomentospora prolificans*	[Blood]		MZ359555	>16	8	>8	>8	>8	>8
JMRC:NRZ:2845	*Lomentospora prolificans*	[Blood]		MZ359554	>16*	8	>8	>8	>8	>8
JMRC:NRZ:2088	*Montagnula opulenta*	Contact lens		MZ382433						
JMRC:NRZ:1439	*Penicillium chrysogenum*	Contact lens (keratitis)		MZ382430						
JMRC:NRZ:1605	*Penicillium citrinum*	Vitreous body		MZ359604						
JMRC:NRZ:2685	*Penicillium crustosum*	Corneal swab	patient C	MZ359605						
JMRC:NRZ:2911	*Penicillium rubens*	Contact lens cleaning solution (keratitis)	patient D	MZ359607						
JMRC:NRZ:1023	*Penicillium rubens*	Corneal swab		MZ359606						
JMRC:NRZ:0182	*Peniophora lycii*	Vitreous body		MZ382425						
JMRC:NRZ:2328	*Peroneutypa scoparia*	Corneal swab		MZ382435						
JMRC:NRZ:1395	*Petriella setifera*	Corneal swab of eye abscess (intraoperative)		MZ382429						
JMRC:NRZ:2869	*Plectosphaerella cucumerina*	Anterior chamber		MZ382443						
JMRC:NRZ:1764	*Plectosphaerella* sp.	Contact lens cleaning solution (keratitis)		MZ382434						
JMRC:NRZ:1568	*Pseudopithomyces* sp.	Cornea		MZ382431						
JMRC:NRZ:0396	*Purpureocillium lilacinum*	Corneal ulcer		MZ359564	>16	>32	0.06	2	0.06	0.125
JMRC:NRZ:0438	*Purpureocillium lilacinum*	(Keratitis)		MZ359565	>16	>32	0.5	>8	0.125	0.5
JMRC:NRZ:0560	*Purpureocillium lilacinum*	(Keratitis)		MZ359566	>16	>32	0.5	>8	0.25	0.5
JMRC:NRZ:0673	*Purpureocillium lilacinum*	Contact lens cleaning solution (keratitis)		MZ359567	>16	>32	2	>8	1	0.5
JMRC:NRZ:1189	*Purpureocillium lilacinum*	Vitreous body		MZ359569	>16	>32	0.125	1	0.5	0.25
JMRC:NRZ:1349	*Purpureocillium lilacinum*	Corneal swab		MZ359570	>16	>32	0.5	>8	0.06	0.25
JMRC:NRZ:1559	*Purpureocillium lilacinum*	(Keratitis)		MZ359571	>16	>32	0.125	1	0.125	0.125
JMRC:NRZ:1591	*Purpureocillium lilacinum*	Corneal scraping		MZ359572	>16	>32	0.06	2	0.125	0.125
JMRC:NRZ:1596	*Purpureocillium lilacinum*	Corneal swab		MZ359573	>16	>32	0.25	4	0.25	0.125
JMRC:NRZ:2624	*Purpureocillium lilacinum*	Corneal scraping		MZ359579	>16	>32	0.25	1	0.06	0.25
JMRC:NRZ:2842	*Purpureocillium lilacinum*	Corneal swab		MZ359580	>16	>32	1	>8	0.5	0.25

JMRC:NRZ:2894	*Purpureocillium lilacinum*	Corneal scraping		MZ359581	>16	>32	0.25	>8	0.06	0.125
JMRC:NRZ:1020	*Purpureocillium lilacinum*	(Keratitis)		MZ359568	>16	>32	1	>8	0.5	0.25
JMRC:NRZ:2067	*Purpureocillium lilacinum*	Corneal scraping		MZ359577	>16	>32	0.5	4	0.06	0.25
JMRC:NRZ:1891	*Purpureocillium lilaciuum*	Corneal scraping		MZ359576	>16	>32	0.5	>8	0.125	0.5
JMRC:NRZ:0148	*Purpureocillium sodanum*	Corneal swab		MZ359561	>16	>32	n.a.	>8	0.5	0.25
JMRC:NRZ:0203	*Purpureocillium sodanum*	Anterior chamber		MZ359562	>16	>32	0.25	8	0.125	0.25
JMRC:NRZ:0245	*Purpureocillium sodanum*	Corneal scraping		MZ359563	>16	>32	0.125	2	0.06	0.125
JMRC:NRZ:1638	*Purpureocillium sodanum*	Cornea		MZ359574	>16	>32	1	>8	0.125	0.25
JMRC:NRZ:1672	*Purpureocillium sodanum*	Vitreous body		MZ359575	>16	>32	0.25	>8	0.125	0.25
JMRC:NRZ:2136	*Purpureocillium sodanum*	Vitreous body		MZ359578	>16	>32	0.5	>8	0.125	0.5
JMRC:NRZ:3040	*Purpureocillium sodanum*	[Tracheal bronchial secretion]		MZ359582	>16	>32	0.5	>8	0.25	0.25
JMRC:NRZ:0647	*Rhinocladiella similis*	Vitreous body		MZ382427						
JMRC:NRZ:1929	*Sarocladium kiliense*	Contact lens cleaning solution	patient E	MZ359619						
JMRC:NRZ:2044	*Sarocladium kiliense*	Corneal scraping		MZ359618						
JMRC:NRZ:1444	*Sarocladium spinificis*	Corneal ulcer		MZ359621						
JMRC:NRZ:1424	*Sarocladium strictum*	Contact lens (endophthalmitis)	patient A	MZ359620						
JMRC:NRZ:0330	*Scedosporium apiospermum*	Cornea		MZ359376	8	2	>8	>8	>8	1
JMRC:NRZ:1504	*Scedosporium apiospermum*	[Sputum]		MZ359382	4	2	8	>8	2	0.5
JMRC:NRZ:1585	*Scedosporium apiospermum*	[Wound. lower leg]		MZ359383	1	2	8	>8	2	1
JMRC:NRZ:1796	*Scedosporium apiospermum*	[Sputum]		MZ359384	8	4	8	4	1	0.5
JMRC:NRZ:1833	*Scedosporium apiospermum*	Corneal scraping		MZ359385	>16	4	8	>8	4	0.5
JMRC:NRZ:1957	*Scedosporium apiospermum*	Infekt der oberen Atemwege		MZ359386	16	2	8	2	2	1
JMRC:NRZ:2230	*Scedosporium apiospermum*	Corneal swab		MZ359388	16	4	>8	>8	>8	1
JMRC:NRZ:2334	*Scedosporium apiospermum*	[Bronchial secretion]		MZ359389	4	2	>8	>8	>8	1
JMRC:NRZ:2531	*Scedosporium apiospermum*	Corneal swab		MZ359390	2	2	>8	>8	>8	1
JMRC:NRZ:2638	*Scedosporium apiospermum*	[Bronchial alveolar lavage]		MZ359393	8	2	>8	>8	>8	0.5
JMRC:NRZ:2863	*Scedosporium apiospermum*	[Knee puncture]		MZ359394	16	2	8	>8	2	0.5
JMRC:NRZ:1100	*Scedosporium apiospermum*	Corneal scraping		MZ359381	2	2	8	4	2	0.5
JMRC:NRZ:2080	*Scedosporium apiospermum*	Corneal scraping		MZ359387	>16	4	>8	>8	4	1
JMRC:NRZ:2558	*Scedosporium apiospermum*	Eye		MZ359391	16	4	>8	>8	>8	1
JMRC:NRZ:2634	*Scedosporium aurantiacum*	[Wound. thumb]		MZ359392	>16	2	8	>8	2	0.5
JMRC:NRZ:0964	*Scedosporium dehoogii*	(Endophthalimitis)		MZ359377	8	2	4	1	0.25	0.25
JMRC:NRZ:2013	*Scedosporium dehoogii*	[Sputum]		MZ359380	16	2	2	1	1	0.5
JMRC:NRZ:2872	*Scedosporium dehoogii*	Corneal scraping		MZ359379	8	2	4	>8	0.5	0.5
JMRC:NRZ:1173	*Scedosporium dehoogii*	[Intraoperative swap of phlegmon of hand]		MZ359378	>16	2	8	1	2	0.5
JMRC:NRZ:2675	*Schizophyllum commune*	Corneal swab	patient C	MZ382440						
JMRC:NRZ:1367	*Scopulariopsis brevicaulis*	Corneal graft		MZ359622	8	4	>8	>8	>8	>8
JMRC:NRZ:2682	*Scopulariopsis brevicaulis*	[Ear]		MZ359623	>16	4	>8	>8	>8	>8

JMRC:NRZ:0706	*Tintelnotia destructans*	Eye		MZ359625	0.5		8	0.5	0.125	1
JMRC:NRZ:2326	*Tintelnotia destructans*	Konjunctiva and contact lens		MZ359627	0.5	2	8	0.5	0.125	1
JMRC:NRZ:2039	*Tintelnotia destructans*	Contact lens (keratitis)		MZ359626	1	1	4	0.25	0.06	0.5
