# Supplementary material for: Eye Infections Caused by Filamentous Fungi: Spectrum and Antifungal Susceptibility of the Prevailing Agents in Germany
Source: J Fungi (Basel). 2021 Jun 26;7(7):511. doi: 10.3390/jof7070511 (PMC8307352; doi:10.3390/jof7070511)
Supplement: Supplementary file 1 [file jof-07-00511-s001.zip › Walther et al._table S2 rev.pdf]

Table S2: Markers amplified for the identification of eye infecting filamentous fungi, primers and annealing temperatures used for PCR

**Genus**	**Gene/DNA region**	**Primers**	**Annealing temperature**	**Reference**
Alternaria	Major allergen (Alt a 1)	Alt-for (5'-ATGCAGTTCACCACCATCGC-3'); Alt-rev (5'-ACGAGGGTGAYGTAGGCGTC-3')	58°C	[1]
Aspergillus (sect. *Fumigati*)	Calmodulin (caM)	cmd5 (5'-AATGAACGACCAATGGAAAAG-3'); cmd6 (5'-CCGATAGAGGTCATAACGTGG-3')	54°C	[2]
Aspergillus (remaining sections), *Penicillium*	Calmodulin (caM)	CL1 (5'-GARTWCAAGGAGGCCTTCTC-3'); CL2A (5'-TTTTTGCATCATGAGTTGGAC-3')	56°C	[3]
Aspergillus, *Chaetomium*, *Penicillium*	β-tubulin (benA, tub-2)	bt2a (5'-GGTAACCAAATCGGTGCTGCTTTC-3'); bt2b (5'-ACCCTCAGTGTAGTGACCCTTGGC-3')	58°C	[4]
Fusarium	Translation elongation factor 1-α (TEF1α)	EF-1 (5'-ATGGGTAAGGAGGACAAGAC-3'); EF-2 (5'- GGAAGTACCAGTGATCATGTT-3')	55°C	[5]
Remaining genera	Nuclear ribosomal internal transcribed spacer (ITS)	V9G (5'-TTACGTCCCTGCCCTTTGTA-3'); LS266 (5'-GCATTCCCAAACAACTCGACTC-3')	54°C	[6,7]

References

1. Hong, S.G.; Cramer, R.A.; Lawrence, C.B.; Pryor, B.M. Alt a 1 allergen homologs from Alternaria and related taxa: Analysis of phylogenetic content and secondary structure. *Fungal Genet. Biol.* **2005**, *42*, 119–129, doi:10.1016/j.fgb.2004.10.009.
2. Hong, S.B.; Cho, H.S.; Shin, H.D.; Frisvad, J.C.; Samson, R.A. Novel Neosartorya species isolated from soil in Korea. *Int J. Syst. Evol. Microbiol.* **2006**, *56*, 477–486, doi:10.1099/ijs.0.63980-0.
3. O'Donnell, K. Molecular phylogeny of the *Nectria haematococca-Fusarium solani* species complex. *Mycologia* **2019**, *92*, 919–938, doi:10.1080/00275514.2000.12061237.
4. Glass, N.L.; Donaldson, G.C. Development of primer sets designed for use with the PCR to amplify conserved genes from filamentous Ascomycetes. *Appl. Environ. Microbiol.* **1995**, *61*, 1323–1330.
5. O'Donnell, K.; Cigelnik, E.; Nirenberg, H.I. Molecular Systematics and Phylogeography of the Gibberella fujikuroi Species Complex. *Mycologia* **1998**, *90*, doi:10.2307/3761407.
6. De Hoog, G.S.; Gerrits van den Ende, A.H. Molecular diagnostics of clinical strains of filamentous Basidiomycetes. *Mycoses* **1998**, *41*, 183–189, doi:10.1111/j.1439-0507.1998.tb00321.x.
7. Masclaux, F.; Gueho, E.; de Hoog, G.S.; Christen, R. Phylogenetic relationships of human-pathogenic Cladosporium (Xylohypha) species inferred from partial LS rRNA sequences. *J. Med. Vet. Mycol.* **1995**, *33*, 327–338, doi:10.1080/02681219580000651.
